# The effect of pregnancy on renal angiomyolipoma; a world of knowledge to gain, specifically in women with TSC

**DOI:** 10.1186/s12882-024-03483-4

**Published:** 2024-03-22

**Authors:** Marlou W. Kluiving, Evelien F. H. I. Peeters, Titia A. Lely, Niek van Oorschot, Wendela L. de Ranitz-Greven

**Affiliations:** 1 Department of Internal Medicine, Center of Expertise for Tuberous Sclerosis Complex, University Medical Center Utrecht, Utrecht, The Netherlands; 2 Department of Obstetrics and Gynecology, University Medical Center Utrecht, Utrecht, The Netherlands; 3 Department of Radiology, St. Antonius Hospital, Nieuwegein, The Netherlands

**Keywords:** Tuberous sclerosis complex, Renal angiomyolipoma, Pregnancy, Haemorrhage, Complications

## Abstract

**Background:**

Women are counseled preconceptionally about the potential risks of rAML progression and chance of complications during and due to pregnancy. However, a systematic search investigating the evidence on which this advice is based does not exist. The aim of this systematic review is to determine the effect of pregnancy on renal angiomyolipoma (rAML) size and risk of haemorrhage in patients with tuberous sclerosis complex (TSC).

**Methods:**

We searched PubMed, EMBASE, Medline and ClinicalTrials.gov using terms for “renal angiomyolipoma” and “pregnancy”. English-language articles published between January 1st 2000, and December 31st 2020 of which full-text was available were included. The initial search resulted in 176 articles. After the screening process we included 45 case reports and 1 retrospective study. For the retrospective study we assessed the risk of bias using the Newcastle–Ottawa Scale. We included articles about renal AML and pregnancy with and without an established diagnosis of TSC. From these articles we recorded the rAML sizes and rAML complications.

**Results:**

Seven case reports, from a total of 45 case reports, provided follow-up data on renal AML size (these were all cases of renal AML without a known diagnosis of TSC). Of these cases, renal AML size decreased in one patient, was stable in one patient, increased in three patients and fluctuated in two others. Renal AML size of women who suffered a haemorrhage were significantly larger (12.1 ± 4.6 cm) than rAMLs of women who did not suffer a haemorrhage (8.3 ± 3.2 cm). Data from the retrospective study showed no difference in renal complications between the women with and without a history of pregnancy. Haemorrhage occurred in 30% of the women with a history of pregnancy (*n* = 20) and in 11% in the patients without a history of pregnancy (*n* = 2), however this retrospective study had methodological limitations.

**Conclusion:**

The effect of pregnancy on renal AML size and complications in patients with TSC is unclear. More research is needed to determine the risk of pregnancy on TSC-associated kidney disease in TSC patient.

**Supplementary Information:**

The online version contains supplementary material available at 10.1186/s12882-024-03483-4.

## Background

Tuberous sclerosis complex (TSC) is an autosomal dominant disease that causes development of benign lesions in multiple organs as a result of dysregulation of the mTOR pathway [1]. Renal angiomyolipoma (rAML) has a high prevalence (60–80%) in adult patients with TSC [1, 2]. Renal AML is a benign tumor that consist of three cell types; smooth muscle, adipose tissue, and vascular endothelial cells [3, 4]. Renal AMLs are often asymptomatic, but complications that can occur are pain, haemorrhage, hypertension [5, 6]. Renal failure may develop after surgical interventions [6]. The TOSCA registry, a large TSC cohort, documented renal hemorrhagic symptoms in 5.4% of patients at baseline, and in 1.6% at the first follow-up visit [7]. The number of patients with TSC that underwent renal embolization or nephrectomy is estimated around 25% [8]. Renal AMLs are more frequently found in women than in men [1, 5]. The exact reason for this remains unclear. However, research has shown that this could be a result of the presence of estrogen receptors on rAML tissue [9, 10]. As estrogen levels increase during pregnancy, it is hypothesized that these receptors may be responsible for faster growth of rAML during pregnancy [9]. As the current criterion to determine the risk of rAML rupture is rAML size and growth, it is thought that pregnancy leads to more rAML ruptures [11]. When the rAML size is greater than 3 cm, the chance of rAML rupture increases [12]. Due to this larger risk, patients with growing rAML above 3 cm are often recommended to start mTOR inhibiting medication or undergo local therapy such as embolization or partial nephrectomy as second line choice of therapy [13].

In current practice, women are counseled preconceptionally about the potential risks of rAML progression and chance of complications during and due to pregnancy. However, a systematic search investigating the evidence on which this advice is based does not exist. As the number of female patients with TSC and pregnancy wish increases, it is important to inform this group accurately of the potential risks. This systematic review study was performed to assess the evidence of growth and increased risk for complications of rAML caused by pregnancy. To examine this, we systematically reviewed the evidence from literature from 2000–2020 on rAML and pregnancy.

## Sources

We completed a systematic search following the PRISMA (Preferred Reporting Items for Systematic Reviews and Meta-Analyses) reporting guidelines [14]. We searched the PubMed, Embase, Medline and ClinicalTrials.gov databases to find English-language studies published between 2000 and 2020. In anticipation of a limited number of studies specifically addressing TSC-related renal angiomyolipoma (rAML), we decided to include all articles on rAML and pregnancy. Despite distinctions, including manifestation at an older age, lower frequency, and slower growth rate for patients with sporadic rAMLs [3], we chose to include these studies, ensuring that the possibility of potentially (partially) extrapolating evidence from sporadic rAMLs to TSC patients is not precluded. The following search strategy, created by two clinical researchers (M.K. and E.P) was used in PubMed, and similarly in Embase, Medline and ClinicalTrials.gov: ("renal angiomyolipoma*" OR "angiomyolipoma of kidney" OR "angiomyolipoma kidney" OR " kidney angiomyolipoma" OR "renal AML*" OR "kidney AML*") AND ("Pregnancy"[MeSH] OR "Pregnancy" OR "Pregnant" OR "Pregnant Women" [MeSH] OR "Pregnant Women"). The review was not registered and a protocol was not prepared.

## Study selection

**Fig. 1:**
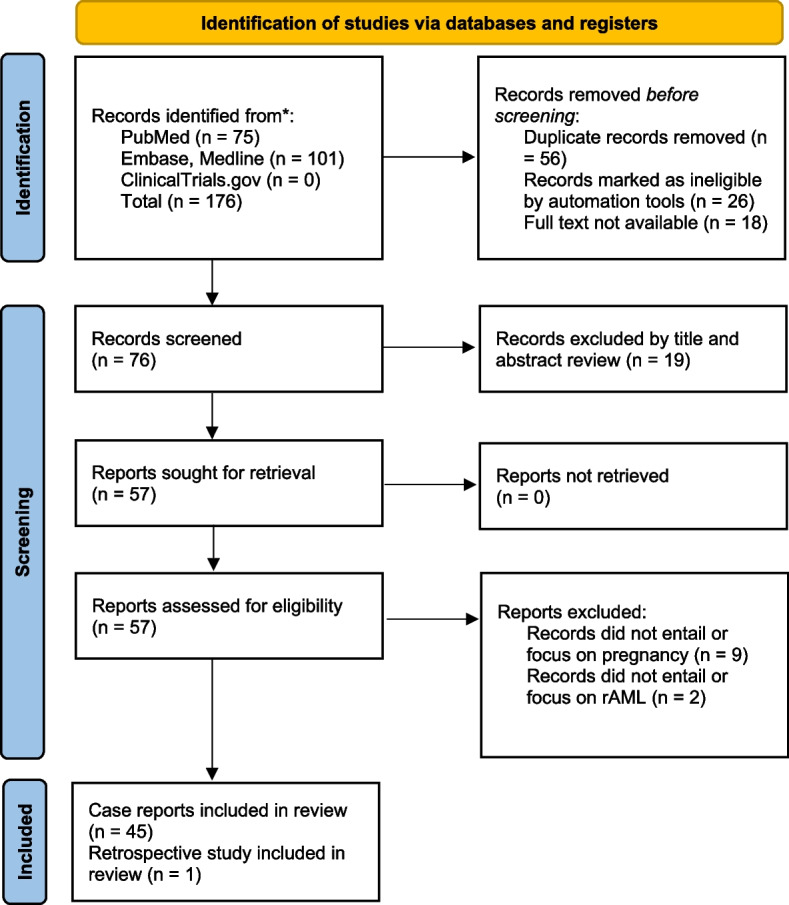
PRISMA (Preferred Reporting Items for Systematic Reviews and Meta-Analyses) flow diagram summarizing the flow of studies in the systematic review

We limited our search to articles published from 1st of January 2000 to 31st of December 2020. Before the screening, we excluded non-English language articles through the automation tools. Only studies with full texts available were considered in this systematic review. Duplicates were removed. The screening process through which we selected the studies was done by one author (M.K.) and is summarized in Fig. 1. In case of uncertainty during the screening process, consultation with the co-author (E.P) was done. We included 46 studies from which data was extracted.

We divided our systemic review in an analysis of the case reports and an analysis of the retrospective study. From all the case reports we extracted the following data: year of publication, patient age, pregnancy history, diagnosis of TSC, date of diagnosis of renal angiomyolipoma, date of first rAML related clinical sign, rAML size before/during/after pregnancy, rAML complication (defined as haemorrhage, renal aneurysm, hypovolemic shock, thromboembolism and growth), treatment (defined as embolization, partial and radical nephrectomy, mTOR inhibitors), case outcome (health status mother and child) and lastly the delivery method (elective or emergency caesarian section or vaginal delivery). Case reports were grouped based on the TSC diagnosis and data of rAML diagnosis. Above-described outcomes are displayed in tables, Table 1 and additional files. The rAML size was reported in a non-standard graph to depict all available rAML measurements and the possible effect of pregnancy. Renal AML complications are shown in a bar graph. Missing data will be explicitly described or traceable by stating the number of patients included. From the retrospective analysis, we extracted information about pregnancy, number of patients with renal involvement, number of women with renal complications and renal hemorrhage. Study quality of the retrospective study was assessed with the Newcastle–Ottawa Scale (NOS) [15]. Two authors (M.K. and E.P.) independently completed the scale. Discrepancies were resolved by the senior author (W.G.).

**Table 1:** Collected data overview (summarized) from the 9 case reports from patients with TSC

Article	Year	Age (yrs.)	Pregnancy history	Diagnosis rAML^a^	rAML size (cm): before pregnancy	rAML size (cm): during pregnancy	rAML size (cm): after pregnancy	rAML complication^b^	Treatment rAML during pregnancy^c^
Clearly Goldman [16]	2004	23	G1P0	Before				NC	CT
Ferianec [17]	2012	30	G1P0	During		L: 21 × 12x8		H	N
Idilman [18]	2014	25	G1P1	After				H (PP)	
Liu [19]	2015	39	G3P0	During (G3)		L: 21.7 R: 21.2		H	E (PP)
Lucky [20]	2009	21	G1P1	Before	L: 12 × 9.5			H (PP)	CT
Ogawa [21]	2013	33	G1P0	Before				NC	
Peces [22]	2011	25	G2P2	After (P2)			R: 7 × 8 L: 14 × 14x11	NC	
Schreider-Monteiro [23]	2003	34		Before		R: 9	R: 20 × 15x16^d^ L: 13.0 × 9.5x5.5^d^	NC	CT
Yamamura [24]	2017	32	G1P0^d^	Before				AG	E, mTORi

*PP* Post-partum, *R* Right, *L* Left, *NC* No complication, *H* Haemorrhage, *AG* rAML growth, *CT* Conservative treatment, *N* Nephrectomy, *E* Embolization

^a^Diagnosis rAML in relation to pregnancy, before/during/after pregnancy

^b^Refers to rAML complications during pregnancy window unless described different

^c^Refers to treatment during pregnancy and treatment directly after induced delivery or emergency caesarean section

^d^AML size measure > 1 year after described pregnancy

^d^Embolization prior to current pregnancy

Summary with more detailed information of all collected data regarding the TSC rAMLs and sporadic rAMLs are present in Additional files 1, 2, 3 and 4

Data were described using means with standard deviations (SD). Statistical testing was performed using χ^2^, t-test, Fisher exact test, with significance at *p* < 0.05. Data was not adjusted for age, severity of rAML before pregnancy and TSC type as this information was not available for majority of the cases. Data were analyzed with IBM SPSS Statistics 26.0.0.1.

## Results

Our search yielded 75 articles from PubMed and 101 from Embase and Medline published between the start of 2000 and the end of 2020. ClinicalTrials.gov did not contain articles about rAML and pregnancy. After exclusions, we included 45 case reports, with 48 cases described, and 1 retrospective study (Fig. 1).

### Case reports

The 45 case reports included data from 48 cases (Additional file 1, 2, 3 and 4). Each case involved a patient with at least one pregnancy. The group consisted of women with an average age of 30.3 (SD 5.5; *n* = 48) years old. The diagnosis TSC was established, based on clinical or genetic diagnostic criteria, in nine of the 48 cases (18.8%) (Table 1, Additional file 1, 2, 3 and 4). From these nine cases, seven women had the TSC diagnosis before the described pregnancy. The other 39 patients were not investigated for TSC, had sporadic rAML or no mention of TSC in their case report. The average rAML size was 11.0 cm (SD 4.6; *n* = 40). From the 48 cases, 8.3% of the women received rAML treatment (embolization (*n* = 4) or/and everolimus (*n* = 1)) before pregnancy.

We divided the 48 cases into four different groups. One group with an evidently established TSC diagnosis (*n* = 9, Table 1, Additional file 1). The other patients (*n* = 39), without TSC diagnosis (not tested, not mentioned, or proven sporadic rAML), were divided into 3 groups based on the moment the presence of rAML became apparent: rAML diagnosis before pregnancy (*n* = 8, Additional file 2), rAML diagnosis during pregnancy (*n* = 28, Additional file 3) and rAML diagnosis after pregnancy (*n* = 3, Additional file 4). The majority of the women (*n* = 41) came to the hospital with complaints related to rAML in the 2nd (43.9%) or 3rd (34.1%) trimester. The complaints were predominantly flank pain and hematuria.

**Fig. 2:**
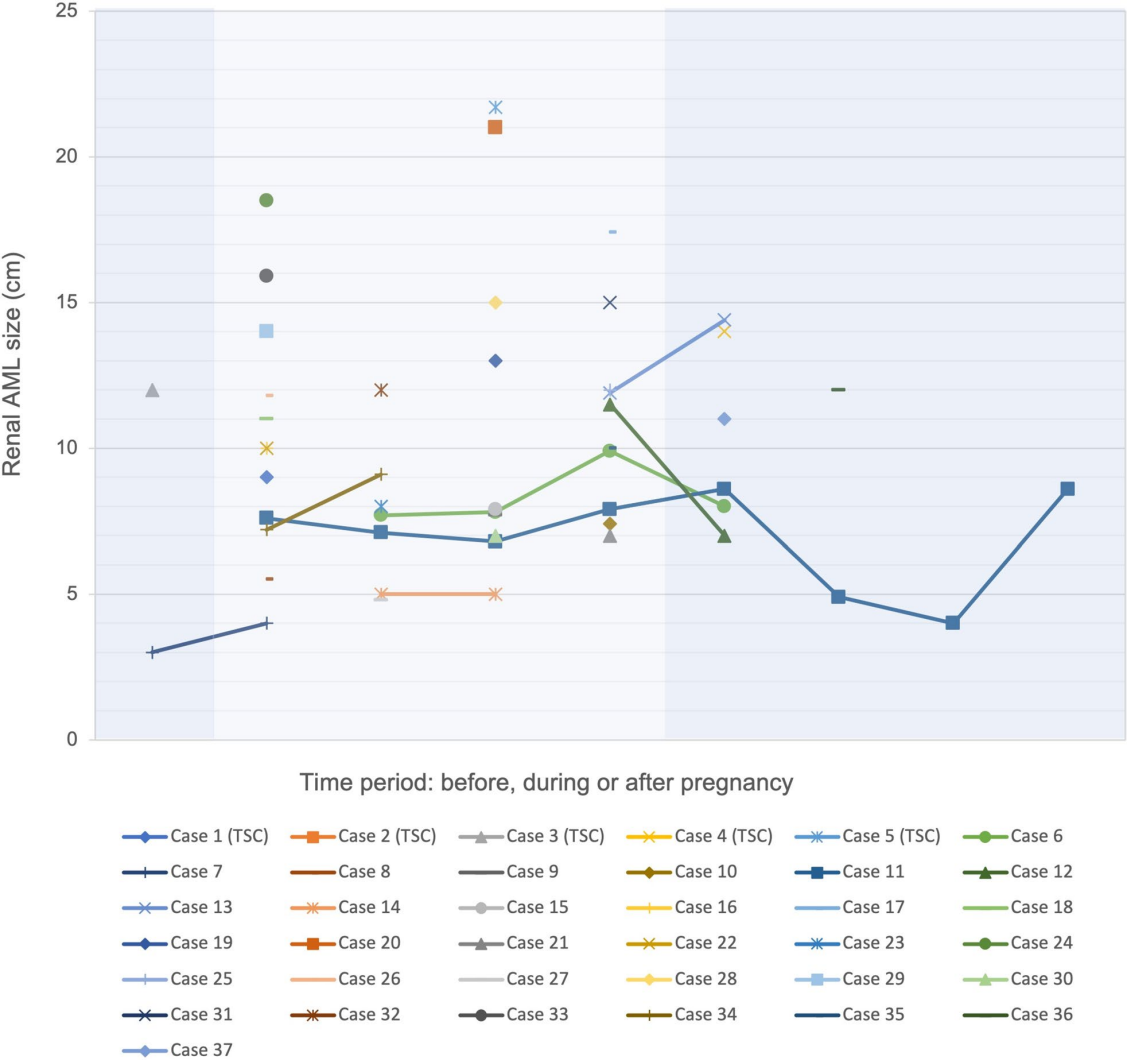
Overview of all rAML measurements before, during and after pregnancy

In order for us to determine whether measured rAML sizes were reliable for interpretation we set up two criteria. Firstly, rAML size measurements had to be collected during pregnancy or one year before or after the pregnancy. This way we limited the change in size caused by time. Secondly, rAML sizes measured after individuals were treated for rAML by embolization or mTOR inhibitor therapy were excluded. Results from computed tomography (CT) scans were chosen over ultrasounds when made on the same day. The 37 cases that met these criteria and had one or more rAML measurements available are illustrated (Fig. 2). Of the 37 cases, seven cases had more than one measurement available, none of which involved a patient with a known TSC diagnosis. The rAML size increased in three cases, decreased in one case, stayed consistent in one case and was variable over time in two cases. From the 37 cases, case number 25 (Fig. 2) depicts a rAML measurement of 3.0 cm before pregnancy. Lopater et al. [25] described the formation of a tumorthrombus in this case. The other 97.3% had a rAML size of 4.0 cm or lager. Renal AMLs of women who suffered a haemorrhage were significantly larger (12.1 ± 4.6 cm) than rAMLs of women who did not suffer a haemorrhage (8.3 ± 3.2 cm; *p* = 0.018). For patients with TSC, rAML sizes were 12 cm [20], 21 cm [17] and 21.70 cm [19] in patients with haemorrhage and 9 cm [23] and 14 cm [22] without haemorrhage.

Only measurements that met the reliability criteria described in the results section were used. We placed the rAML measurement in chronological order on the x-axis when there was more than one measurement available in one-time frame. Location of a single rAML measurement was placed randomly in the time frame. This allowed us to make each rAML size visible, as there was overlap between rAML sizes among different cases. In case rAML was bilaterally we chose the value that had a follow up rAML measurement. When this was not available, we chose the largest rAML measurement. Case numbers 1–5 display patients in which TSC has been established.

**Fig. 3:**
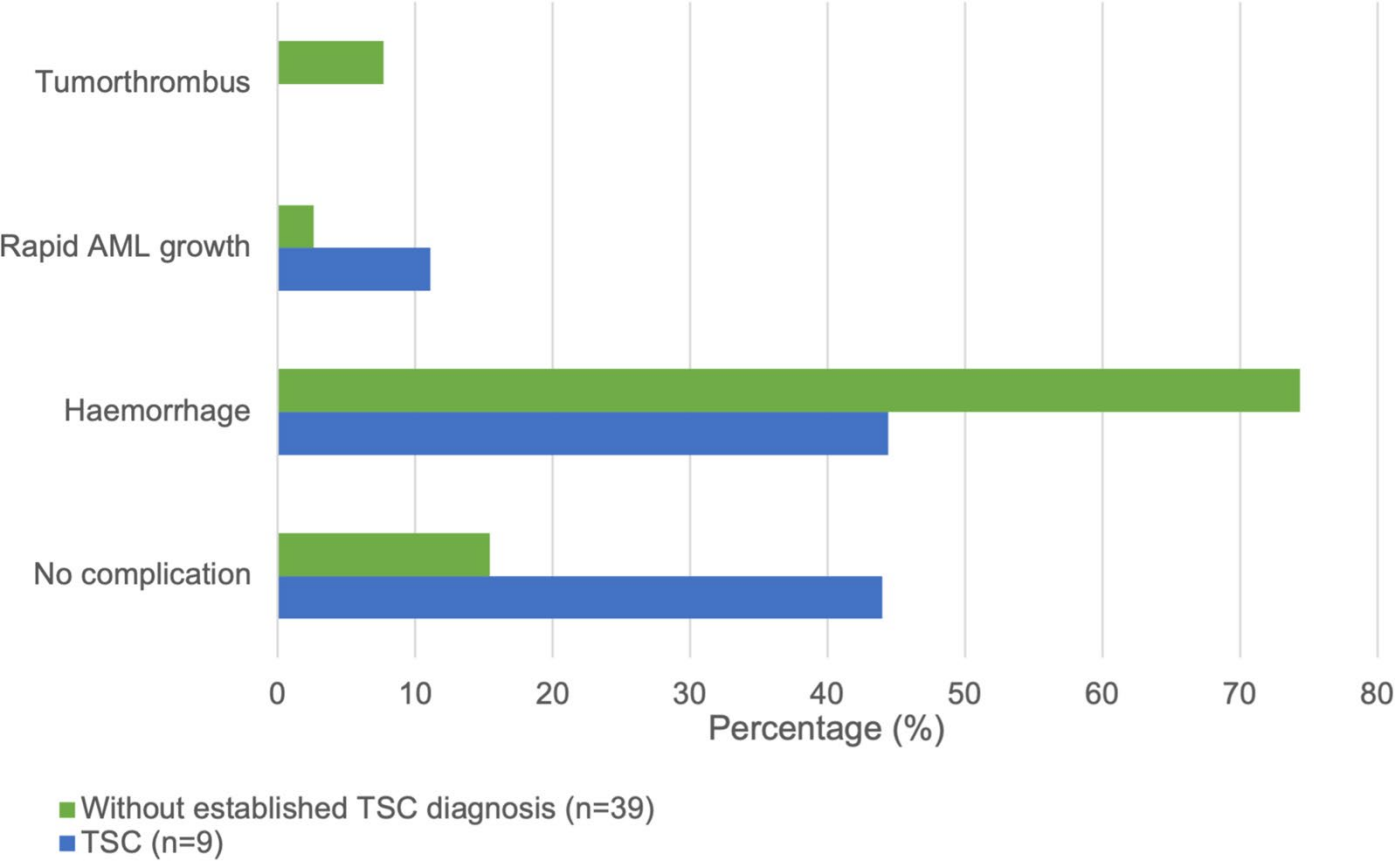
Renal complication occurrences, described in the included case reports, in percentages (%) in patients with (*n* = 9) and without (*n* = 39) an established TSC diagnosis. No patients with TSC developed a tumorthrombus. Rapid rAML growth was only noted when explicitly stated in the article

In Fig. 3, we illustrate the differences in complication rates between patients with and without TSC diagnosis in the 48 cases. Complications (defined as haemorrhage, rapid rAML growth, or tumorthrombus) occurred in 84.7% of patients without TSC diagnosis and in 55.5% of patients with established TSC (*p* = 0.074). Haemorrhage occurred in 44.4% (*n* = 4) of the patients with established TSC compared to the 74.4% (*n* = 29) in the group without diagnosis of TSC (*p* = 0.115). Three patients developed a tumorthrombus, none were diagnosed with TSC (two sporadic rAML, one TSC diagnosis not mentioned in article). Though being a benign entity, rAML can be locoregionally aggressive and show vascular invasion resulting in tumorthrombus formation. This is a rare but known complication of rAML, occurring mostly in large rAMLs (> 4 cm) [26]. The tumorthrombus can grow and extend into the renal vein, possibly all the way to the inferior vena cava (IVC) and right atrium. This is associated with increased risk of (large) pulmonary embolism and also potentially results in IVC obstruction, which can be life threatening conditions for both mother and fetus in pregnancy. Imaging is important for identification of the rAML, determination of the extent of tumorthrombus formation and pre-operative planning, with magnetic resonance imaging (MRI) being the modality of choice during pregnancy. All three patients with tumorthrombi included in this review were treated surgically during pregnancy with either nephrectomy or thrombectomy.

### Retrospective study

We used the study from Mitchell et al. [27] only for analysis of complication risk since data about rAML growth was not provided. The quality assessment score of this study using the NOS was three out of nine, meaning a high risk of bias when translated to the Agency for Health Research and Quality (AHRQ) standards. The study consisted of 145 patients with TSC, 115 with a pregnancy history and 30 without a pregnancy history. This single center study was retrospective and used self-reported data from surveys (response rate was 30%) and data from electronical charts. The average age at which information was obtained was 41.1 (SD = 11.1) years old in the pregnant group and 35.1 (SD = 9.3) years old in the group who had never been pregnant. Renal involvement (rAML or renal cysts) was around 70% in both groups (pregnant and non-pregnant TSC women). Severity or type of renal involvement was not described. Of the women with renal involvement (18 in the non-pregnant group and 67 in the pregnant group) an analysis showed that renal complications were similar in the non-pregnant group, 67%, in comparison to the pregnant group, 57% (*p* = 0.62). Renal complications were defined in this study as either rAML-related hypertension, pain, rupture, haemorrhage, renal failure or rAML-related treatments. Haemorrhage occurred in 30% (*n* = 20) of the pregnant group and in 11% (*n* = 2) of the never-pregnant group (significance not reported). Explanation for omitting the *p*-value could not be found. The rates of haemorrhage in the pregnant group only includes patients with haemorrhage that occurred during or after pregnancy. During pregnancy there were eight cases of haemorrhage which occurred during pregnancy or within five weeks of post-partum. Overall, Mitchell et al. [27] concluded that “pregnancy did not appear to increase either the prevalence of renal involvement or the risk of a renal complication in the women we studied”.

## Discussion

How should women with TSC be counselled preconceptionally? This systematic review shows a significantly larger rAML size in the group with renal haemorrhage. This finding justifies the current criteria to use size as an important factor for haemorrhage risk [28]. However, there were insufficient rAML measurements described within the included case reports to determine the effect of pregnancy on rAML growth. Consequently, the association between pregnancy and rAML complications could not be derived from the case reports. The retrospective study, however, shows no statistically significant difference in renal complications between the pregnant versus never-been pregnant group in patients with TSC and renal involvement.

Eble [29] suggested in 1998 that hormones might play an important role in the growth of rAML during pregnancy. Estrogen receptor expression on rAML made this theory more plausible [9]. Even though guidelines do not recommend health professionals to counsel patients regarding the risk of pregnancy and/or exogenous estrogen use [30], in current practice this is frequently done based on the possible role of estrogen on rAML proliferation [9]. Yu et al. [31] confirmed this role, while Bertolini [32] showed no effect of estrogen on rAML cell growth. These contradictory results illustrate the need for more research to determine the causation between presence of estrogen receptors on rAML and growth/complications during pregnancy or from oral anti-contraceptives.

This is the first study that systematically reviews literature on rAML growth due to or during pregnancy in patients with TSC. We found that there is currently insufficient data to determine the effect of pregnancy on rAML size. Only seven case reports described multiple measurements, which did not show a consistent trend in size change during pregnancy. In addition, none of these cases involved patients with an established TSC diagnosis. Despite the hypothesized differences between TSC related rAMLs and sporadic rAMLs, [3, 9, 33], our study showed no significant differences in rAML complications and haemorrhage between patients with established TSC and patients without an established TSC diagnosis. However, uncertainties about the validity of this conclusion should be acknowledged, considering the study's limitations. Therefore, it remains uncertain whether a comparison between the behavior of rAMLs in women with TSC and those with sporadic rAMLs is justified.

Limitations of this study are that data on rAML growth were based on case reports, which have a high risk of publication bias leading to publication of primarily rare cases. This is stressed by the reported sizes of rAML in the case reports, wherein 90% exceeded a size of 5.0 cm. This in contrast with published rAML sizes in a large cohort of TSC patients (351 patients), which showed that more than half of the patients (around 40 years of age) have renal AML sizes smaller than 3.5 cm [34]. These findings support the hypothesis of reporting bias. In addition, the group of patients that were not diagnosed with TSC also included patients that were not tested for TSC. The possible diagnostic misclassification weakens the observed results. Also, in the included studies, rAML measurements were obtained through various imaging modalities, including ultrasound, MRI, and CT scans. We did not distinguish between these modalities. Notably, 2D scan methods are prone to error, making volumetric analysis the preferred, more accurate approach. Standardizing these methods is crucial for enhancing comparability in future studies. Furthermore, the single retrospective study that was found had a low response rate of 30%. Also, due to the absence of reported rAML sizes, a notable disparity between the pregnant and non-pregnant cohorts within this study cannot be ruled out. Moreover, the age differences between the pregnant versus never-been pregnant group, as well as the unknown degree of rAML severity before pregnancy weaken the results from the retrospective study.

In conclusion, based on current available data there is no compelling reason for health professionals to unduly alarm patients with TSC about the risk of pregnancy on rAML growth or renal complications. A retrospective study analyzing and interpreting all available imaging records, such as CT or MRI scans, and patients’ electronic health records/database in a large cohort of pregnant and non -pregnant TSC patients could contribute to more reliable and meaningful results. These results can offer crucial insights and improve guidance for women with TSC considering pregnancy. Additionally, the collection of risk factors and data, including epidemiologic, pathologic, and imaging evidence, could help the development of a not yet existing risk assessment system proposed by Wang et al. [11]. Such a model would be a useful tool as it could help predict the risk of rAML complications, such as rupture risk, for each patient individually. Lastly, more knowledge regarding pregnancy outcomes in patients with TSC is needed. Unfortunately, most of the case reports in this systematic review did not contain information concerning pregnancy outcomes. However, gaining insight into, for example, gestational age, birth weight, percentage of prematurity and childbirth delivery methods is also relevant for optimizing pregnancy counseling in patients with TSC.

### Supplementary Information

**Additional file 1.**

**Additional file 2.**

**Additional file 3.**

**Additional file 4.**

**Additional file 5.**

## Data Availability

All data generated or analysed during this study are included in this published article [and its supplementary information files].

## Abbreviations

rAML: Renal Angiomyolipoma
TCS: Tuberous Sclerosis Complex
PRISMA: Preferred Reporting Items for Systematic Reviews and Meta-Analyses
NOS: Newcastle-Ottawa Scale
SD: Standard Deviation
CT: Computed Tomography
IVC: Inferior Vena Cava
MRI: Magnetic Resonance Imaging
AHQ: Agency for Health Research and Quality
