# Supplementary material for: The effect of pregnancy on renal angiomyolipoma; a world of knowledge to gain, specifically in women with TSC
Source: BMC Nephrol. 2024 Mar 22;25:113. doi: 10.1186/s12882-024-03483-4 (PMC10960455; doi:10.1186/s12882-024-03483-4)
Supplement: Supplementary file 1 — Additional file 1. [file 12882_2024_3483_MOESM1_ESM.docx]

| Article | Year | Age patient | Pregnant history (GPM) | TSC | Diagnosis rAML^*^ | First Clinical Sign renal AML (GW) | Size rAML (cm) before pregnancy | Size rAML (cm) during pregnancy | Size rAML (cm) after pregnancy | Complication^†^ | Treatment AML during pregnancy^‡^, GW | Delivery method, GW | Pregnancy outcome | Treatment after pregnancy | Size rAML (cm)after rAML treatment |
| --- | --- | --- | --- | --- | --- | --- | --- | --- | --- | --- | --- | --- | --- | --- | --- |
| Clearly Goldman [16] | 2004 | 23 | G1P0 | Yes | Before | - | - | Kidney sizes: R**:** 19 L:20; multiple hypoechoic masses | - | No complication | Conservative management | Elective caesarean section, GW 34 | Healthy child and HS mother | - | - |
| Ferianec [17] | 2012 | 30 | G1P0 | Yes | During | 9 | - | L: 21×12×8^¶^ | - | Haemorrhage | Urgent laparotomy for nephrectomy L, GW 9 | Medical termination pregnancy after treatment, - | HS mother | - | L: 21×12×8^¶^ |
| Idilman [18] | 2014 | 25 | G1P1 | Yes | After | 2 wks. post-delivery | - | - | - | Haemorrhage + Aneurysm | - | -, full term | Healthy child and HS mother | Embolization | - |
| Liu[19] | 2020 | 39 | G3P0 | Yes | During (G3) | 16 | - | L: 21.7  R: 21.2 | - | Haemorrhage + Aneurysm + Hemodynamically unstable + abscess (post-partum) | Embolization, directly after delivery | Medical termination pregnancy, - | HS mother, later abscess developed | Drainage abscess R | - |
| Lucky [10] | 2009 | 21 | G1P1 | Yes | Before | 5hrs post-delivery | Multiple bilateral AMLs; L: 12×9.5 | - | - | Haemorrhage post-partum | Conservative treatment | - | Healthy child, HS mother | Embolization | Decrease in size^\|\|^ |
| Ogawa [21] | 2013 | 33 | G1P0 | Yes | Before | - | Multiple bilateral AMLs | - | Evident increase in bilateral renal cysts and nodules. | No complication | - | - | Healthy child and HS mother | - | - |
| Peces [22] | 2011 | 25 | G2P2 | Yes | After (P2) | 14 | - | - | R: 7×8 L: 14×14×11, 6×4 | No complication | - | Elective caesarean section, GW 38 | Healthy children and HS mother | Embolization L/R + nephrectomy L, + kidney sparing lumbotomy L | R: 12.92×5.73×4.03^\|\|^ R: 14.0×9.0×4.0^¶^ + 3.5×2.5 ^¶^ |
| Schreider-Monteiro [23] | 2003 | 34 | - | Yes | Before | 20 | - | R: 9 | 7 years later:  R: 20x15x16^§^ L: 13×11.5×6.5^§^ | No complication | Observation, follow up after delivery | - | - | Partial nephrectomy (laparotomy) | R: 23.0×21.0×11.5^¶^ L: 13.0×9.5×5.5^¶^ |
| Yamamura [24] | 2017 | 32 | G1P0 | Yes | Before | - | Kidney sizes^\|\|^  R: 18.7  L: 22.3 | Kidney sizes^\|\|^ GW 17 R: 20.5 L: 24.5. GW 22 R: 19.8 L: 23.4. | - | Rapid AML growth | Embolization (GW21, GW24) and everolimus (GW25) of different dosages. | - | Healthy child and HS mother | - | - |

**Additional file 1** Overview and Characteristic of patients with TSC from the included studies.

* Diagnosis AML in relation to pregnancy, before/during/after pregnancy.

† Complication during pregnancy window.

‡ Refers to treatment during pregnancy and treatment directly after induced delivery or emergency caesarean section.

§ AML size measure >1 year before or >1 year after described pregnancy.

|| Measurement rAML size after embolization.

¶ Measurement rAML size after removal (ex vivo).

GW = gestational week, HS = hemodynamically stable R= right, L = left. The dash sign (-) refers to not available data.
