# Supplementary material for: The effect of pregnancy on renal angiomyolipoma; a world of knowledge to gain, specifically in women with TSC
Source: BMC Nephrol. 2024 Mar 22;25:113. doi: 10.1186/s12882-024-03483-4 (PMC10960455; doi:10.1186/s12882-024-03483-4)
Supplement: Supplementary file 2 — Additional file 2. [file 12882_2024_3483_MOESM2_ESM.docx]

| Article | Year | Age patient | Pregnant history (GPM) | TSC | Diagnosis rAML^*^ | First Clinical Sign renal AML (GW) | Size rAML (cm) before pregnancy | Size rAML (cm) during pregnancy | Size rAML (cm) after pregnancy | Complication^†^ | Treatment AML during pregnancy^‡^, GW | Delivery method, GW | Pregnancy outcome | Treatment after pregnancy | Size rAML (cm) after rAML treatment |
| --- | --- | --- | --- | --- | --- | --- | --- | --- | --- | --- | --- | --- | --- | --- | --- |
| Al Ateeqi [28] | 2007 | 29 | G4P2 | No | Before | 32 | L: 6.6.00: 4.9×3.9×3.6  6.5.00: 8.6×4.6×5.5 25.7.01: 8.6×4.6×5.5 | L: 07.02.04: 7.3×6×7.7,  06.07.04: 7.8×7.7×6.7,  21.07.04: 9.9×6.8×6.1 | L:16.08.04: 8.0×6.1 | Hemorrhage (hypovolemic shock) | Conservative treatment till hemodynamically unstable. Cystoscopy and laparoscopic, directly after delivery | Emergency caesarean section, GW 36 | Healthy children and HS mother | Laparoscopic nephrectomy (3 months post-partum) | L: 9×5×5^¶^ |
| Govednik [4] | 2011 | 30 | G3P2 | No | Before | 20 | R: 7.5 (after G2, before embolization)^§^ | Significant growth^§^ of the AML within the right renal vein over a period of 2 months | - | Tumorthrombus | right radical nephrectomy and removal of AML from the right renal vein and vena cava, 2nd trimester | Vaginal delivery, full term | Healthy child and HS mother | - | Multiple AMLs 0.3 - 8.0^¶^. |
| Lopater [25] | 2011 | 34 | G1P0 | No | Before | - | R: 3 | R: 4 | R: 3 | Tumorthrombus | Thrombectomy of the floating AML thrombus in the right venal vein, GW 30 | Elective caesarean section, GW 39 | Healthy child and HS mother | Partial nephrectomy R | R: 3 |
| Mishra [35] | 2016 | 24 | G2P0 | No | Before | - | - | R: 5.5×3.3 Kidney size:  R: 15.17.3 | - | No complication | - | - | Healthy child and HS mother | - | - |
| Ng [36] | 2018 | 33 | G1P0 | No | Before | 32 | R: 2.3^§^ | R: 7.7 | - | Hemorrhage (hypovolemic shock) | Conservative treatment till mother hemodynamically unstable. Embolization (directly after delivery) | Emergency caesarean section, - | Healthy child and HS mother | - | R: 3.5×3.8×2.5^\|\|^ |
| Ng [36] | 2018 | 34 | G2P1 | No | Before | - | R: 3.5×3.8×2.5^\|\|^ | - | R: 3.5×3.8×2.5^\|\|^ | No complication | - | Elective caesarean section, - | Healthy child and HS mother | - | - |
| Storm [37] | 2006 | 32 | - | No | Before | 39 | L: 7.0×4.1×8.0^\|\|^  R: 5.5×1.7×5.0^\|\|^ | L: 5.6x6.8 hyperechoic region with blood flow^\|\|^ | No change in size of left sided lesion. | Hemorrhage | Conservative treatment | Vaginal delivery, full term | Healthy child and HS mother | - | - |
| Zhang [38] | 2020 | 27 | G1P0 | No | Before | - | - | R: 7.4×5.3×4.7 (GW 24) | - | Tumorthrombus | Retroperitoneal laparoscopic nephrectomy R with open tumor thrombectomy, GW 26 | Spontaneous vaginal delivery, GW 39 | Healthy child and HS mother | - | - |

**Additional file 2** Overview and characteristic of patients that were diagnosed with rAML before pregnancy from the included studies.

* Diagnosis AML in relation to pregnancy, before/during/after pregnancy.

† Complication during pregnancy window.

‡ Refers to treatment during pregnancy and treatment directly after induced delivery or emergency caesarean section.

§ AML size measure >1 year before or >1 year after described pregnancy.

|| Measurement rAML size after embolization.

¶ Measurement rAML size after removal (ex vivo).

GW = gestational week, HS = hemodynamically stable R= right, L = left. The dash sign (-) refers to not available data.

REFERENCES

4. Govednik-Horny C, Atkins M. Angiomyolipoma With Vascular Invasion During Pregnancy. Annals of Vascular Surgery 2011;25(8):1138.e9-13. doi: 10.1016/j.avsg.2011.05.023

25. Lopater J, Hartung O, Bretelle F, Bastide C. Management of angiomyolipoma vena cava thrombus during pregnancy. Obstetrics Gynecology 2011;117(2):440-443. doi: 10.1097/AOG.0b013e3181edbc56

28. Al-Ateeqi A, Ali RH, Kehinde EO, Mujaibel K, Al-Hunayan A, Al-Harmi J. Increasing severity of haematuria with successive pregnancies in a woman with renal angiomyolipoma. Int Urol Nephrol 2007;39(2):409-12. doi: 10.1007/s11255-006-9036-2

35. Mishra VV, Mistry K, Nanda S, Choudhary S, Gandhi K. Successful pregnancy outcome in a patient with solitary kidney affected by angiomyolipoma: A rare case. Journal of Clinical and Diagnostic Research 2016;10(10):6-7. doi: 10.7860/JCDR/2016/21448.8670

36. Ng T, Chu RW, Leung CL, Chan WK, Cho C, Low I, et al. Spontaneous rupture of renal angiomyolipoma during pregnancy: A report of two cases and literature review. Surgical Practice 2018;22(4):185-191. doi: 10.111/1744-1633.12314

37. Storm DW, Mowad JJ. Conservative management of a bleeding renal angiomyolipoma in pregnancy. Obstetrics Gynecology 2006;107(2):490-2. doi: 10.1097/01.AOG.0000167392.59472.c3

38. Zhang Z, Liu Z, Yao L, Zhang Q, He Z. Surgical management of angiomyolipoma with vena cava thrombus during pregnancy: A case report. Translational Andrology and Urology 2020;9(2):807-811. doi: 10.21037/tau.2019.12.43
