# Supplementary material for: The effect of pregnancy on renal angiomyolipoma; a world of knowledge to gain, specifically in women with TSC
Source: BMC Nephrol. 2024 Mar 22;25:113. doi: 10.1186/s12882-024-03483-4 (PMC10960455; doi:10.1186/s12882-024-03483-4)
Supplement: Supplementary file 3 — Additional file 3. [file 12882_2024_3483_MOESM3_ESM.docx]

| Article | Year | Age patient | Pregnant history (GPM) | TSC | Diagnosis rAML^*^ | First Clinical Sign renal AML (GW) | Size rAML (cm) before pregnancy | Size rAML (cm) during pregnancy | Size rAML (cm) after pregnancy | Complication^†^ | Treatment AML during pregnancy^‡^, GW | Delivery method, GW | Pregnancy outcome | Treatment after pregnancy | Size rAML (cm) after rAML treatment |
| --- | --- | --- | --- | --- | --- | --- | --- | --- | --- | --- | --- | --- | --- | --- | --- |
| Abrams [5] | 2011 | 32 | G1P0 | No | During | 32 | - | L: 7.9×6.9×6.2 | - | Haemorrhage + Aneurysm | Embolization, - | -, full term | - | - | L: 3.2^\|\|^ |
| Al Ateeqi [28] | 2007 | 26 | G3P1 | No | During (3rd pregnancy) | 12 | - | L: 8.8.99: 6.8×4.1×3.6, 24.7.99: 7.1×4.5×3.1, 22.7.99: 7.6×4.2×3.2, 6.10.99: 7.9×3.4×3.5 | L: 6.5.00: 8.6×4.6×5.5,  6.6.00: 4.9×3.9×3.6, 18.6.00: 4×3, 25.7.01: 8.6×4.6×5.5 | No complication | - | Vaginal delivery, - | - | Refused treatment | - |
| Ao [39] | 2017 | 30 | G1P0 | No | During | 20 | - | L: Lesion 1: 10  Lesion 2: 1.1 | - | Haemorrhage + Aneurysm | Conservative treatment till GW 34. Embolization (directly after delivery). | Emergency caesarean section, GW 34 | Healthy child and HS mother | - | Lesion 1: 9^\|\|^ |
| Binkowska [40] | 2009 | 26 | - | No | During | 20 | - | L:  12.0×12.4×17.4  + two small (< 1 cm) lesions | - | Haemorrhage + Aneurysm + Hemodynamically unstable | Embolization, - | Elective caesarean section, GW 38 | Healthy child and HS mother | Total nephrectomy L | No growth^\|\|^ |
| Çetin [41] | 2015 | 26 | G1P0 | No | During | 34 | - | L: 11×6.5×5.5 | - | No complication | Conservative treatment | Induced vaginal delivery, - | Healthy child and HS mother | Laparoscopic radical nephrectomy (2wks post-partum) | - |
| Davis [42] | 2013 | 35 | G3P0 | No | During | 16 | - | R: 13×11×11  L: 8.5x8.5x8 | R: decrease in size^\|\|^, L: decrease in size | Haemorrhage + Pseudoaneurysm | Embolization, - | Elective caesarean section, GW 38 | Healthy child and HS mother | - | - |
| Gao [43] | 2019 | 31 | G2P0 | No | During | 20 | - | L: Lesion 1: 3.0×3.0×2.0  Lesion 2: 21×12×10 | - | Haemorrhage | NephrectomyL and splenectomy (emergency laparotomy) | - | Delivery of a non-fetal fetus (3days post-surgery) and HS mother | Another tumor resection surgery was performed | - |
| Góes Junior [44] | 2019 | 33 | - | No | During | 34 | - | L: 10×6  R: 0.8×0.6 | - | Haemorrhage | Conservative treatment | Elective caesarean section, - | Healthy child and unstable mother | Emergency embolization (4days post-partum) | - |
| Gyimadu [45] | 2011 | 21 | - | No | During | 25 | - | L: 11.5×9.5  R: 3.5 | L: 7  R: 3 | Haemorrhage | Conservative treatment | Elective caesarean section, GW 38 | Healthy child and HS mother | Embolization L | - |
| Iruloh [46] | 2013 | 23 | G1P0 | No | During | 31 | - | L: 11.9×9.8 with haemorrhage within the mass | L: 14.4×12 | Haemorrhage + Aneurysm | Conservative treatment | Elective caesarean section, GW 38 | Healthy child and HS mother | Embolization + later a nephrectomy | Growth^\|\|^ |
| Kira [47] | 2016 | 30 | - | No | During | 19 | - | L: 12 | - | Hemorrhage | Conservative treatment till GW34. Embolization (directly after delivery) | Emergency caesarean section, GW 34 | Healthy child and HS mother | - | - |
| Kontos [48] | 2008 | 28 | G1P0 | No | During | 33 | - | R: 7×7×5 | - | Hemorrhage (hypovolemic shock) | Conservative treatment till fetal distress. Nephrectomy R (directly after delivery) | Emergency caesarean section due to fetal distress, - | Healthy child and HS mother | - | - |
| Morales [49] | 2005 | 28 | - | No | During | 10 | - | Mass of mixed echogenicity: R: 10×7×6 | - | Haemorrhage | Embolization, GW 12) | Vaginal delivery, GW 40 | Healthy child and HS mother | - | - |
| Myoen [50] | 2015 | 37 | - | No | During | 25 | - | R: 8 | - | Ceased haemorrhage + Aneurysm | Embolization, GW 25 | Elective caesarean section, GW 37 | Healthy child and HS mother | Embolization (5 months post-partum) | - |
| Nath [51] | 2018 | 30 | G1P0 | No | During | 32 | - | R: 18.5×11.5 | - | Hemorrhage (hypovolemic shock) | Radical nephrectomy, - | Emergency caesarean section, GW 32 | Healthy child and HS mother | - | - |
| Ng [36] | 2018 | 36 | - | No | During | 9 | - | L: 12 | - | Hemorrhage (hypovolemic shock) | Embolization, - | Elective caesarean section, GW 39 | Healthy child and HS mother | - | L: 5.9×5.3×6.9^\|\|^ |
| Nicola [52] | 2007 | 37 | G1P0 | No | During | 15 | - | R: 11.8×9.9×8.4 | - | Hemorrhage (hypovolemic shock) | Nephrectomy, - | - | Healthy child and HS mother | - | - |
| Pontis [53] | 2013 | 33 | G2P0 | No | During | 34 | - | L: 4.8×4.6 | - | Hemorrhage (hypovolemic shock) | Nephrectomy (directly after delivery) | Emergency caesarean section, GW 34 | Healthy child and HS mother | - | - |
| Preece [54] | 2015 | 45 | G6P1 | No | During | 24 | - | R: 15×14×13 | - | Haemorrhage + Pseudoaneurysm | Conservative till hemodynamically instable. Embolization + Radical nephrectomy R, GW 24 | Elective caesarean section, GW 38 | Healthy child and HS mother | - | - |
| Rana [55] | 2016 | 24 | G1P0 | No | During | 6 | - | - | - | Haemorrhage | Embolization (not successful), GW6. Partial nephrectomy with evacuation of hematoma, GW6 | Elective caesarean section, - | Healthy child and HS mother | - | - |
| Lopes dos Santos [56] | 2014 | 40 | G3P2 | No | During | 18 | - | R: 5  R (+ 2 mnd): 5 | - | Haemorrhage | Conservative treatment | Elective caesarean section, GW 35 | Healthy child and HS mother | Refused treatment and follow-up postpartum | - |
| Scharf [57] | 2019 | 30 | G2P0 | No | During | 6 | - | R: 14×11×12 | - | Haemorrhage | Embolization, 1st trimester | - | - | - | - |
| Sen [58] | 2013 | 24 | G1P0 | No | During | 3rd trimester | - | - | - | Haemorrhage + Aneurysm | Conservative treatment | Elective caesarean section, - | Healthy child and HS mother | Embolization + Nephrectomy | L: 20×16×15^\|\|^  L: 22×27× 20^¶^ |
| Tanaka [59] | 2001 | 23 | G2P1 | No | During | 27 | - | L: 7 | - | Hemorrhage | Conservative treatment | Induced vaginal delivery and vacuum extraction, GW38 | Healthy child and HS mother | Embolization (7 days post-partum) | - |
| Tsili [60] | 2017 | 41 | - | No | During | 7th month | - | - | R: 12×8×4.5 | No complication | - | Premature birth due to fetal complications | HS mother | Radical nephrectomy R | - |
| Tupikowski [61] | 2014 | 25 | G2P1 | No | During | 16 | - | R:7.2×3.3 cm (ultrasound @GW 16) R:9.1×7.0 cm (MRI @GW 25) | - | Rapid AML growth (polycystic nephroblastoma could not be excluded as potential diagnosis) | Radical nephrectomy R (directly after delivery) | Emergency caesarian section, GW 29 | Child with mild cerebral palsy and HS mother (histopathology revealed AML) | Nephrectomy | - |
| Ugwumba [62] | 2016 | 26 | G2P1 | No | During | 14 | - | R: 15×12 cm | - | Hemorrhage | Radical nephrectomy, GW 14,5 | Elective caesarian section, GW 40 | Healthy child and HS mother | - | - |
| Zhang [63] | 2020 | 38 | G2P1 | No | During | 19 | - | L:15.9×10 cm R:3.0×2.5 cm | - | Hemorrhage | Nephrectomy L (directly after delivery) | Induction labor, - | - | - | - |

**Additional file 3** Overview and characteristic of patients that were diagnosed with rAML during pregnancy from the included studies.

* Diagnosis AML in relation to pregnancy, before/during/after pregnancy.

† Complication during pregnancy window

‡ Refers to treatment during pregnancy and treatment directly after induced delivery or emergency caesarean section.

|| Measurement rAML size after embolization.

¶ Measurement rAML size after removal (ex vivo).

GW = gestational week, HS = hemodynamically stable R= right, L = left. The dash sign (-) refers to not available data.

REFERENCES

5. Abrams J, Yee DC, Clark TWI. Transradial embolization of a bleeding renal angiomyolipoma. Vascular and Endovascular Surgery 2011;45(5):470-3. doi: 10.1177/1538574411408352

28. Al-Ateeqi A, Ali RH, Kehinde EO, Mujaibel K, Al-Hunayan A, Al-Harmi J. Increasing severity of haematuria with successive pregnancies in a woman with renal angiomyolipoma. Int Urol Nephrol 2007;39(2):409-12. doi: 10.1007/s11255-006-9036-2

36. Ng T, Chu RW, Leung CL, Chan WK, Cho C, Low I, et al. Spontaneous rupture of renal angiomyolipoma during pregnancy: A report of two cases and literature review. Surgical Practice 2018;22(4):185-191. doi: 10.111/1744-1633.12314

39. Ao L, Ogasahara E, Okuda Y, Hirata S. Spontaneous rupture of renal angiomyolipoma during pregnancy. BMJ Case Reports 2017;2017. doi: 10.1136/bcr-2016-217284

40. Binkowska M, Debska M, Mazurek M, Slapa R, Debski R. Embolization of renal angiomyolipoma in pregnancy: case report. Ginekol Pol 2009;80(6):449-52.

41. Çetin C, Büyükkurt S, Demir C, Evrüke C. Renal angiomyolipoma during pregnancy: Case report and literature review. Turkish Journal of Obstetrics and Gynecology 2015;12(2):118-121. doi: 10.4274/tjod.32848

42. Davis NF, Kelly R, Lee MJ, Mohan P. Selective arterial embolisation of bilateral angiomyolipomata in a symptomatic pregnant female. BMJ Case Reports 2013;2013. doi: 10.1136/bcr-2013-009256

43. Gao CM, Ma YQ, Yu C, Xie N, Ma Y. A case report of giant hamartoma of both kidneys with spontaneous rupture and hemorrhage in a pregnant woman: A case report. Biomedical Reports 2019;11(2):59-62. oi: 10.3892/br.2019.1223

44. Góes Junior AMO, Jeha SAH, De Oliveira CP, Dos Santos DAS. Embolization of Ruptured Renal Angiomyolipoma in Puerpera. Revista brasileira de ginecologia e obstetricia : revista da Federacao Brasileira das Sociedades de Ginecologia e Obstetricia 2019;41(3):199-202. doi: 10.1055/s-0039-1683948

45. Gyimadu AO, Kara A, Basaran D, Esinler I. Conservative management of a retroperitoneal hemorrhage following a ruptured renal angiomyolipoma in pregnancy. J Obstet Gynaecol Res 2011;37(2):156-9. doi: 10.1111/j.1447-0756.2010.01326.x

46. Iruloh C, Keriakos R, Smith DJ, Cleveland T. Renal angiomyolipoma and lymphangioleiomyomatosis in pregnancy. Journal of Obstetrics and Gynaecology 2013;33(6):542-6. doi: 10.3109/01443615.2013.812622

47. Kira S, Sawada N, Miyamoto T, Mitsui T, Zakoji H, Takeda M. Hemorrhagic Renal Angiomyolipoma in Pregnancy Effectively Managed by Immediate Cesarean Section and Elective Transcatheter Arterial Embolization: A Case Report. Journal of Endourology Case Reports 2016;2(1):65-67. doi: 10.1089/cren.2016.0030

48. Kontos S, Politis V, Fokitis I, Lefakis G, Koritsiadis G, Simaioforidis V, et al. Rapture of renal angiomyolipoma during pregnancy: a case report. Cases Journal 2008 -10-17;1(1):245. doi: 10.1186/1757-1626-1-245

49. Morales JP, Georganas M, Khan MS, Dasgupta P, Reidy JF. Embolization of a Bleeding Renal Angiomyolipoma in Pregnancy: Case Report and Review. Cardiovasc Intervent Radiol 2005;28(2):265-268. doi: 10.1007/s00270-004-1850-2

50. Myoen S, Mitsuzuka K, Saito H, Ota H, Takase K, Arai Y. Spontaneous rupture of a renal angiomyolipoma at 25 weeks of pregnancy treated with transarterial embolization: A case report and review of the literature. International Journal of Urology 2015;22(7):710-2. doi: 10.1111/iju.12775

51. Nath S, Sadhu S, Dhar D, Nadar D, Wadhawan M, Varshney P. Rupture of renal angiomyolipoma during pregnancy: A case report. Journal of Anaesthesiology Clinical Pharmacology 2018;34(2):280-281. doi: 10.4103/joacp.JOACP_293_17

52. Nicola M, Gulfi G, Milanese S, De Luca F. Spontaneous rupture of renal angiomyolipoma in pregnancy at 15 weeks gestation. Archivio Italiano di Urologia, Undrologia 2007;79(4):179-80.

53. Pontis A, Piras B, Meloni A, De Lisa A, Melis GB, Angioni S. Rupture of renal angiomyolipoma in pregnancy. Journal of Obstetrics and Gynaecology 2013;33(6):628-9. doi: 10.3109/01443615.2013.810201

54. Preece P, Mees B, Norris B, Christie M, Wagner T, Dundee P. Surgical management of haemorrhaging renal angiomyolipoma in pregnancy. International Journal of Surgery Case Reports 2015;7:89-92. doi: 10.1016/j.ijscr.2015.01.004

55. Rana MA, Mady AA, Jakaraddi N, Mumtaz SA, Ahmad H, Naser K. Not All Acute Abdomen Cases in Early Pregnancy Are Ectopic; Expect the Unexpected: Renal Angiomyolipoma Causing Massive Retroperitoneal Haemorrhage. Case Reports in Critical Care 2016;2016:5643470. doi: 10.1155/2016/5643470

56. Lopes dos Santos MM, Proença SMS, Pereira de Almeida Reis, M I N, Viana RMAL, Martina LMB, Dos Reis Colaço JM, et al. Spontaneous rupture of renal angiomyolipoma during pregnancy. Revista Brasileira de Ginecologia e Obstetrícia 2014;36(8):377-80. doi: 10.1590/so100-720320140005019

57. Scharf Z, Momah-Ukeh I, Kim AY. Trans-Radial Embolization of Bleeding Renal Angiomyolipoma in Pregnant 30-Year-Old Female - A Case Report. Journal of radiology case reports 2019;13(2):34-42. doi: 10.3941/jrcr.v13i2.3511

58. Sen JPB, Murphy K, Patterson JM, Smith DJ. Management of expanding giant renal angiomyolipoma in pregnancy. Journal of Clinical Urology 2014;7(1):63-65. doi: [10.1177/2051415813490](https://doi.org/10.1177/2051415813490153)153

59. Tanaka M, Kyo S, Inoue M, Kojima T. Conservative management and vaginal delivery following ruptured renal angiomyolipoma. Obstet Gynecol 2001;98(5):932-3. doi: 10.1016/s0029-7844(01)01489-2

60. Tsili AC, Ntorkou A, Argyropoulou MI. Renal Epithelioid Angiomyolipoma Associated with Pulmonary Lymphangioleiomyomatosis: Imaging Findings. Journal of Clinical Imaging Science 2017;7(18). doi: 10.4103/jcis.JCIS_14_17

61. Tupikowski K, Bialy A, Dembowski J, Zlotkiewicz M, Guziński M, Kolodziej AK, et al. Kidney angiomyolipoma in pregnancy. Central European journal of urology 2014;66(4):434-6. doi: 10.5173/ceju.2013.04.art11

62. Ugwumba FO, Nnakeny EF, Okafor OC, Onuh AC, Ezechukwu PC, Urube S. Renal angiomyolipoma in pregnancy: Surgical management with fetal preservation - Approach in a developing setting. Clinics and Practice 2016;6(4):105-107. doi: 10.4081/cp.2016.893

63. Zhang T, Xue S, Wang ZM, Duan XM, Wang DX. Diagnostic value of ultrasound in the spontaneous rupture of renal angiomyolipoma during pregnancy: A case report. World Journal of Clinical Cases 2020;8(17):3875-3880. doi: 10.12998/wjcc.v8.i17.3875
