# Supplementary material for: The effect of pregnancy on renal angiomyolipoma; a world of knowledge to gain, specifically in women with TSC
Source: BMC Nephrol. 2024 Mar 22;25:113. doi: 10.1186/s12882-024-03483-4 (PMC10960455; doi:10.1186/s12882-024-03483-4)
Supplement: Supplementary file 4 — Additional file 4. [file 12882_2024_3483_MOESM4_ESM.docx]

| Article | Year | Age patient | Pregnant history (GPM) | TSC | Diagnosis rAML* | First Clinical Sign renal AML (GW) | Size rAML (cm) before pregnancy | Size rAML (cm) during pregnancy | Size rAML (cm) after pregnancy | Complication^†^ | Treatment AML during pregnancy^‡^ | Delivery method, GW | Pregnancy outcome | Treatment after pregnancy | Size rAML (cm) after rAML treatment |
| --- | --- | --- | --- | --- | --- | --- | --- | --- | --- | --- | --- | --- | --- | --- | --- |
| Zapardiel [64] | 2010 | 30 | G1P0 | No | After | 35 | - | - | L: 11 | Haemorrhage (hypovolemic shock) | Embolization (same day as delivery) | Emergency cesarean section due to fetal distress, GW 35 | Healthy child and HS mother | - | - |
| Liu [65] | 2015 | 31 | G1P1 | No | After | 37 | - | - | - | - | - | Emergency cesarean section due to fetal distress, GW 37 | Healthy child and HS mother | Emergency nephrectomy (hemodynamically unstable, 3 days post-partum) | R: 8×6 |
| Orywal [66] | 2015 | 31 | G3P3 | No | After | Few hours after delivery | - | - | - | Hemorrhage (hypovolemic shock) | - | - | - | Embolization (1day post-partum) | - |

**Additional file 4** Overview and characteristic of patients that were diagnosed with rAML after pregnancy from the included studies.

* Diagnosis AML in relation to pregnancy, before/during/after pregnancy.

† Complication during pregnancy window

‡ Refers to treatment during pregnancy and treatment directly after induced delivery or emergency/elective caesarean section.

GW = gestational week, HS = hemodynamically stable R= right, L = left. The dash sign (-) refers to not available data.

REFERENCES

64. Zapardiel I, Delafuente-Valero, J, Bajo-Arenas, J M. Renal angiomyolipoma during pregnancy: review of the literature. Gynecol Obstet Invest 2011;72(4):217-9. doi: 10.1159/000329328

65. Liu J, Meng T, Yang X, Zhao G, Li B. Spontaneous rupture of renal angiomyolipoma in the third trimester. Taiwanese Journal of Obstetrics and Gynecology 2015;54(6):788-90. doi: 10.1016/j.tjog.2015.10.016

66. Orywal AK, Zeilie M, Brüning R, Gross AJ, Netsch C. Rupture of renal angiomyolipoma during childbirth. Urology 2015;85(4):19-20. doi: 10.1016/j.urology.2014.12.008
